# Decreasing incidence of ruptured abdominal aortic aneurysm already before start of screening

**DOI:** 10.1186/s12872-016-0215-5

**Published:** 2016-02-17

**Authors:** Sofia Nessvi Otterhag, Anders Gottsäter, Bengt Lindblad, Stefan Acosta

**Affiliations:** Vascular Center, Institution of Clinical Sciences, Lund University, SE-205 02 Malmö, Lund, Sweden

**Keywords:** Ruptured abdominal aortic aneurysm, Incidence, Population-based study, Autopsy, Screening

## Abstract

**Background:**

The aim of this study was to evaluate whether screening for abdominal aortic aneurysm (AAA) has led to a decrease in ruptured AAA (rAAA) incidence.

**Method:**

The Malmö population was evaluated regarding the incidence of rAAA and elective AAA surgery 4 years before and after start of AAA-screening in 2010. Data from 1971 to 1986 (J Vasc Surg 18:74–80, 1993) and 2000–2004 (J Vasc Surg 44:237-43, 2006), enabled analysis of trends over time.

**Results:**

Analysis of time-periods 1971–1986, 2000–2004, 2006–2010 and 2010–2014 showed an incidence of rAAA of 5.6 (4.9–6.3), 10.6 (8.9–12.4), 6.1 (4.6–7.6) and 4.0 (2.9–5.1), respectively. In men aged 60–69 years the incidences were 16.0 (10.7–21.3), 45.6 (27.7–63.4), 19.3 (9.2–35.3) and 8.9 (2.8–20.6), respectively. The incidences of elective AAA surgery in men aged 60–69 years were 22.9 (16.5–29.2), 34.6 (19.1–50.2), 9.7 (1.2–18.5) and 44.2 (27.0–61.6), respectively.

**Conclusions:**

A decrease in incidence of rAAA in men was evident before the implementation of screening. We were yet not able to demonstrate a certain reduction in rAAA incidence after the start of screening.

## Background

Abdominal aortic aneurysm (AAA) mainly affects men and is a potentially life threatening condition due to the risk for aneurysm rupture (rAAA) [1]. A substantial portion of rAAA patients dies outside hospital [2]. Since AAA is generally asymptomatic before rupture, early detection of the disease is of great clinical importance, and this has been made possible by the introduction of large scale screening programs.

The benefit of screening programs has been supported by four large randomized controlled trials (RCTs) [3–6], in populations from 1988 to 1999 when AAA prevalence rates in 65-year old men ranged between 3.5 and 4.5 %. These studies showed a 40 % relative reduction in AAA related death in males [7]. In the MASS study [6], there was a 0.14 % absolute risk reduction in AAA related death (0.19 % in the screened group and 0.33 % in the control group). However, more recent studies now indicate a decreasing prevalence of AAA with rates between approximately 1–2 % [8–11], and the previously demonstrated outcomes of AAA screening should be viewed in this context. Simultaneously, improvements in the surgical management of AAA repair has led to superior outcomes [12], compared to previous studies [3–6].

The aim of the present study was to evaluate the incidence of rAAA and elective AAA surgery, in Malmö, Sweden, before and after introduction of ultrasound AAA screening of men aged 65 years in September 2010. Analysis of trends over time was enabled by results from previously published data from 1971 to 1986 [13] and 2000–2004 [14].

## Methods

### Study population

This study comprises the population of Malmö 4 years before and 4 years after start of screening for AAA in men aged 65 years, in September 2010. Between 2006 and 2014, the population of Malmö increased from 276,244 to 318,107 (Swedish Central Bureau of Statistics). The populations in 2008 (286,535) inhabitants and 2012 (307,758 inhabitants) were used as the reference populations. The study was approved by the Regional Ethical Review Board in Lund, Sweden.

The evaluation of rAAA incidence alterations over time was possible due to two previous published studies on AAA incidences in Malmö. The population in 1978 (240,000 inhabitants) was used as reference population in the first study [13] (autopsy frequency 85 %; Bengtsson et al. 1993), between 1971 and 1986. The second study [14] (autopsy frequency 25 %; Acosta et al. 2006) was carried out between 2000 and 2004, with a reference population of 265,481 inhabitants in 2002.

Malmö has one hospital, Skåne University Hospital. During the 8 year study period (September 6, 2010 to September 6, 2014), all surgical procedures and all diagnoses assigned to patients upon discharge or death were classified according to the International Classification of Disease, 10th edition (ICD-10) code and collected in a computerized registry. Data on mortality and domicile was obtained by record linkage with the Swedish Population Registry.

Adherence to the AAA screening program in men aged 65 years, in the population of Malmö, was 80.2 % [15].

### Retrieval of rAAA cases

rAAA was defined as either an extravasation of blood or hematoma outside the AAA -, a diagnosis made with computed tomography (CT), or a hematoma found outside the AAA at open or endovascular aneurysm repair (EVAR), or at autopsy. The ICD-10 code I71.3 was used for identification of patients with rAAA. The Vascular Centre Malmö-Lund is a tertiary referral centre for patients with vascular disease, patients who lived outside Malmö were excluded from this analysis, however.

### Retrieval of clinical and forensic autopsy-verified cases of rAAA

The clinical autopsies were performed according to a standardized detailed protocol at the Department of Clinical Pathology Skåne University Hospital, Lund. Average autopsy rates were 19 % between 2006 and 2010 and 12 % between 2010 and 2014. Findings were coded according to the Systematized Nomenclature of Medicine (SNOMED), as defined by the College of American Pathologists. The topographic code for aorta (t42) and the code of diagnosis for aneurysm (m324) or rupture (m1443) were used in a search to identify all individuals with rAAA. A computerized autopsy registry was used to identify patients with rAAA.

Forensic autopsies were performed at the Institution of Forensic Medicine, Lund, upon request from police authorities. A search to identify individuals with rAAA was performed using the code for aortic aneurysm and dissection (441) before 2013 (according to the ICD-9-CM diagnostic codes) and the codes for diagnoses rAAA (I71.3) and ruptured thoracic aneurysm (171.1) after 2013 was used (according to ICD-10-CM diagnostic codes).

### Evaluation of elective and acute AAA surgery

Elective surgery for an AAA was indicated, in the absence of significant comorbidity, if maximal AAA diameter on ultrasound was >5.0 cm (>5.5 cm in men or >5.0 cm in women measured at CT scan). Acute surgery was performed in ruptured AAA. EVAR was the first choice method for both elective and acute AAA repairs between 2006 and 2014.

Patients from Malmö who underwent elective surgery for AAA or acute surgery for rAAA were identified (ICD code I71.4 and I71.3 respectively and codes for open repair [OR] or endovascular aneurysm repair [EVAR]). All patient records were retrieved for verification of the diagnosis.

### Statistical methods

Age- and gender-specific incidence rates were based on the number of cases of rAAA or elective AAA repairs, respectively, and were expressed as number of cases per 100 000 person-years. Age- and gender- specific event rates and incidence rates from 2006 to 2010 and 2010 to 2014 were computed using the 2008 and 2012 populations in Malmö, respectively, as reference, and 95 % confidence intervals (CIs) were calculated assuming a Poisson distribution of events, using the exact method for *N* < 15 and the normal approximation for larger numbers.

Age in the figures was presented as median (interquartile range [IQR]). Differences in proportions were evaluated using the Chi [2] test. Mann–Whitney U tests were used when comparing groups using continuous variables. *P* < 0.05 was considered significant.

## Results

### Elective repair of AAA

Elective EVAR was performed in 74 (97 %) patients during 2006–2010 and 84 (97 %) Malmö city patients during 2010–2014. Open repair was performed in two and three patients, respectively. The total incidences of elective AAA surgery were 6.6 (5.1–7.7) and 7.0 (5.5–8.5) (per 100 000 person years [95 % CI]), respectively. The ratio of elective versus acute AAA surgery increased from 1.5 (76/50) during 2006–2010 to 2.8 (87/23) during 2010–2014 in the whole population, and from 1.5 (58/38) to 4.4 (74/17) in males and 1.5 (18/12) to 2.2 (12/6) in females, respectively. (Table 1).

**Table 1:** Epidemiology of abdominal aortic aneurysm (AAA) in Malmö during 1971–1986 [13], 2000–2004 [14], 2006–2010 and 2010–2014

Study	Bengtsson et al.	Acosta et al.	Before start of screening	After start of screening
	1971–1986	2000–2004	2006–2010	2010–2014
Demographic data				
Malmo Population	240 000	265 000	285 514	307 207
Percentage > 75 years	6 %	10 %	9 %	8 %
Ratio F:M in persons > 75 years	2:1	1:9:1	3:2	3:2
Autopsy frequency	85 %	25 %	19 %	12 %
Incidence of rAAA^a^				
Men	8.4 (7.1–9.7)	18.1(14.8–21.4)	10.5 (7.9–13.1)	6.2 (4.2–8.2)
60–69 years	16.0 (10.7–21.3)	45.6 (27.7–63.4)	19.3 (9.2–35.3)	8.9 (2.8–20.6)
70–79 years	55.8 (42.7–68.9)	117 (84.3–149.0)	75.4 (45.2–105.6)	44.4 (21.9–66.9)
80+ years	113 (75.9–150) 3.0	154 (102–206)	99.8 (57.2–142.4)	86.4 (46.52–126.3)
Women	3.0 (2.2–3.8)	3.6 (2.2–5.1)	2.9 (2.3–3.5)	1.9 (0.8–3.0)
60–69 years	0.8 (0.1–3.6)	6.5 (1.8–16.6)	7.2 (2.0–18.4)	0 (0–6.2)
70–79 years	8.6 (4.5–12.7)	11.5 (4.6–23.7)	14.1 (5.2–30.8)	7.1 (1.5–21.7)
80+ years	43.0 (28.3–57.7)	25.1 (12.5–44.9)	16.5 (6.6–34.0)	22.3 (10.1–42.4)
All	5.6 (4.9–6.3)	10.6 (8.9–12.4)	6.1 (4.6–7.6)	4.0 (2.9–5.1)
Surgically treated/all rAAA(%)cases				
Men	52/155 (34)	62/116 (53)	38/59 (64)	17/38 (45)
Women	9/60 (15)	5/25 (20)	12/17 (71)	6/12 (50)
All	61/215 (28)	67/141 (48)	50/76 (66)	23/50 (46)
Incidence of elective AAA surgery^a^				
Men	5.9 (4.7–7.0)	10.1 (7.7–12.6)	10.3 (7.6–13.0)	12.8 (9.9–15.6)
60–69 years	22.9 (16.5–29.2)	34.6 (19.1–50.2)	9.7 (1.2–18.5)	44.2 (27.0–61.6)
70–79 years	33.5 (23.4–43.6)	67.7 (43.1–92.3)	113.1(76.1–150.1)	88.8 (57.0–120.6)
80+ years	12.5 (3.4–32.1)	68.1 (33.6–102)	99.8 (57.1–142.1)	91.2 (50.2–132.2)
Women	1.1 (0.6–1.6)	4.1 (2.6–5.6)	3.1 (1.7–4.5)	1.9 (0.8–3.0)
60–69 years	2.8 (1.1–5.8)	12.9 (5.6–25.5)	5.4 (1.1–15.8)	3.3 (0–7.8)
70–79 years	5.1 (2.4–9.3)	27.9 (14.6–41.1)	14.1 (5.2–30.1)	16.7 (4.4–29.0)
80+ years	1.3 (0.0–7.3)	4.6 (0.6–16.5)	18.9 (8.0–37.1)	5.0 (0–11.8)
All	3.4 (2.8–4.0)	7.0 (5.6–8.4)	6.6 (5.1–7.7)	7.0 (5.5–8.5)
Ratio (N/N) elective/acute surgery				
Men	2.1 (108/52)	1.0 (65/62)	1.5 (58/38)	4.4 (75/17)
Women	2.4 (22/9)	5.6 (28/5)	1.5 (18/12)	2.0 (12/6)
All	2.1 (130/61)	1.4 (93/67)	1.5 (76/50)	2.8 (87/23)

^a^Incidence per 100,000 person years (95 % CI)

The median age of patients undergoing elective AAA surgery, 4 years before start of screening was 75 years (IQR 71–81), and decreased to 73 years (68–79) 4 years after start of screening (*p* = 0.028). The proportions of males were 73 % (58/76) and 86 % (75/87), respectively (*p* = 0.104). The incidence of elective AAA surgery in men aged 60–69 years was 22.9 (16.5–29.2) in 1971–1986 [13] (Bengtsson et al. 1993), 34.6 (19.1–50.2) in 2000–2004 [14] (Acosta et al. 2006), and decreased significantly to 9.7 (1.2–18.5) in 2006–2010, and then after start of screening increased significantly to 44.2 (27.0–61.6) in 2010–2014 (per 100 000 person years [95 % CI]). (Table 1).

### Epidemiology of rAAA

In 2006–2010 and 2010–2014 the diagnosis was established after clinical autopsy in 16 (21 %) and 16 (36 %) patients, respectively, whereas 5 (7 %) and 4 (9 %), respectively, had been referred from the police authorities to the Institution of Forensic Medicine (Table 2). Autopsy rates decreased from 85 % in 1971–1986, to 25 % in 2000–2004, to 19 % in 2006–2010, and finally to 12 % in 2010–2014 (Table 1, Fig. 2).

**Table 2:** Clinical characteristics of rAAA patients in 2000–2004 [14], 2006–2010 and 2010–2014

Study	Acosta et al.	Before start of screening	After start of screening	*P*-value
	2000–2004	2006–2010	2010–2014	
	N (%)	N (%)	N (%)	
Age				
- 59	3 (2)	4 (5)	0	
60–69	29 (21)	14 (19)	5 (10)	
70–79	57 (41)	30 (40)	18 (36)	
80+	51 (36)	28 (37)	27 (54)	0.168
Gender				
Male	115 (82)	59 (78)	38 (76)	0.565
Autopsy				
Clinical	54 (40)	16 (21)	16 (36)	0.104
Forensic	9 (6)	5 (7)	4 (9)	
Deaths from rAAA	103 (74)	43 (57)	35 (70)	0.014
Total	140 (100)	76 (100)	50 (100)	-

During the 4 year time periods before and after start of screening in September 2010, 76 versus 50 rAAA patients were identified, respectively. The median age of rAAA patients was 76 (IQR 70–83) years in 2006–2010 and 80 (75–85) years in 2010–2014 (*p* = 0.071), and the proportions of patients aged 80 years or older were 28 (37 %) and 27 (54 %), respectively. The proportions of men suffering from rAAA during the respective time period, were 59 (78 %) and 38 (76 %). Before and after start of screening 43 (57 %) out of 76 and 35 (70 %) out of 50 persons suffering from rAAA died (*p* = 0.014) (Table 2). During the 4 year time periods before and after start of screening, in total four men and one man, respectively, aged 65–69 years died from rAAA. In the respective time periods two and 12 65–69-year old men underwent elective AAA surgery, and none died within 30 days of elective surgery. The absolute risk for AAA related mortality 4 years before and after implementation of screening was 1.3 % (4/308 [AAA related mortality/total mortality of 65–69 year old men]) and 0.3 % (1/300), respectively. This corresponds to a relative risk reduction of 75 %.

**Fig. 1:**
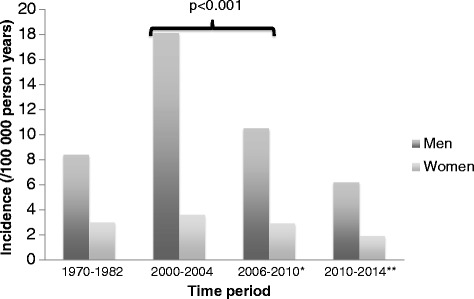
Total population incidence of ruptured abdominal aortic aneurysm in Malmö between 1970 and 2014. *September 2006 – September 2010. Before start of screening. ** September 2010 – September 2014. After start of screening

**Fig. 2:**
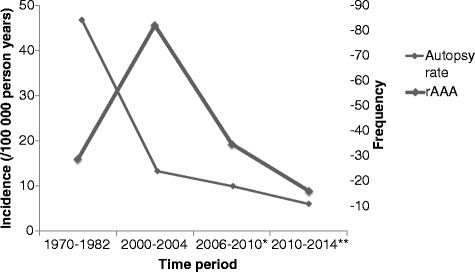
Autopsy rate and incidence of ruptured abdominal aortic aneurysm (rAAA) in men, between 60 and 69 years of age, in Malmö. *September 2006 – September 2010. Before start of screening. ** September 2010 – September 2014. After start of screening

The overall incidences of rAAA were 6.1 (95 % CI 4.6–7.6) per 100 000 in 2006–2010 and 4.0 (2.9–4.1) in 2010–2014. An analysis of trend over the time-periods showed a significant increase in the total incidence of rAAA per 100 000 person years in men during 1971–1986 [13] (Bengtsson et al. 1993) and 2000–2004 [14] (Acosta et al. 2006); from 8.4 (7.1–9.7) to 18.1 (14.8–21.4), and then significant decreases in 2006–2010 to 10.5 (7.9–13.1), and and 2010–2014 to 6.2 (4.2–8.2) (per 100 000 person years [95 % CI]), respectively (Fig. 1). No corresponding decrease in rAAA incidence was seen among women, with incidences of 3.0 (2.2–3.8), 3.6 (2.2–5.1), 2.9 (2.3–3.5), and 1.9 (0.8–3.0) (per 100 000 person years [95 % CI]), respectively. In men aged 60–69 years the incidences of rAAA were 16.0 (10.7–21.3), 45.6 (27.7–63.4), 19.3 (9.2–35.3), and 8,9 (2.8–20.6), respectively (per 100 000 person years [95 % CI]) (Fig. 2, Table 1)

### Management of rAAA patients

Out of the 59 patients in 2006–2010 and 25 in patients 2010–2014 rAAA patients in hospital care for rAAA, 37 (63 %) and 17 (68 %), respectively, were initially managed by vascular or general surgeons and 50 (85 %) and 23 (92 %), respectively, underwent aneurysmal repair. Thus, in-hospital death, without surgery, occurred in nine (15 %) and two (8 %) patients, respectively. In 2006–2010 five out of nine patients were assessed in time and then not considered for surgery: The reasons for non-operative management was high age in three patients, heart failure and severe chronic obstructive disease in the remaining two. During 2010–2014 advanced age was the reason for non-operative management in the one patient assessed in time and not considered for surgery. Five patients between 2006 and 2014 were diagnosed either by clinical examination or CT scan, but died before surgery was possible.

### Screening

During the 4 year period after start of screening in September 2010, a total of 12 (20 %) elective AAA surgeries was performed as a result of aneurysm detection through screening of Malmö city individuals. Two (4 %) patients who suffered from rAAA had previously been subjected to AAA screening (the maximal transverse aortic aneurysm diameter was 7.5 and 5.0 cm, respectively), one of these patients underwent acute surgery for rAAA. Both patients were considered for elective repair, one patient was on the waiting list for surgery and the other was denied repair because of co-morbidities.

## Discussion

The present study is unique, as it is based on the Malmö city population during four decades, and takes the findings in both clinical and forensic post mortem examinations as well as the current autopsy rates into account. Aneurysm rupture is fatal in 74–90 % of cases [2, 14, 16, 17], and about 32 % die outside hospital [2]. Accurate information on prehospital mortality and non-intervention rates in rAAA has been scarce in recent studies [2]. The high average autopsy frequency in the Malmö population between 1971 and 1986 [13] allowed a thorough approximation of the incidence of rAAA. With the decreasing autopsy frequency in recent decades, the diagnostic insecurity rises [18] and generates increased risk of bias towards an underestimation of the true incidence of rAAA [19–21].

In comparison with the other time frames, the peak of total incidence rates of rAAA between 2000 and 2004, might partly be explained by the higher proportion of elderly. This study demonstrates a reduction in the overall incidence of rAAA in men during the last decade, even before start of AAA screening in the autumn of 2010. This finding is in concordance with previous studies, showing both decreasing rAAA incidence and AAA prevalence during the 21st century [2, 8, 22]. This might be explained by improved control of risk factor for AAA development, including improved antihypertensive and lipid lowering treatment. Most importantly, smoking causes 75 % of AAA cases in the population [8, 23] and is the single most important modifiable risk-factor for AAA development. It has been shown that active smoking increases the risk of aneurysm rupture [16, 24, 25] and that the continuous trend of decreasing smoking prevalence [8, 26, 27] amongst age-groups at risk of aneurysm rupture is an essential contributing factor to the decreasing incidence of rAAA. In Sweden, daily smoking among 65–74 year-old men has decreased from 32 % in 1980 to 12.5 % in 2013 [28].

In the age group of men subjected to screening, we found no significant reduction in the incidence of rAAA. Encouraging results from four large randomized screening trials [3, 4, 6, 29], randomizing male population in 1980–1999 with AAA prevalence rates of 4–7.5 %, have led to the implementation of national screening-programs. With the decreasing AAA prevalence rates, the benefit of AAA screening of all men has been questioned, however. The U.S Preventive Services Task Force recommends screening for all 65-year old men who have ever smoked [30]. Another question that needs further investigation is the appropriate age for screening. A study by Svensjö et al. [8] found only a 1.7 % prevalence of screening-detected AAA in 65-year-old men in Sweden. Additionally, with an increased life expectancy [28], patients have a longer lifespan after screening, during which they are at risk for late AAA development. Studies have also indicated a shift in AAA disease to affect older patients [8, 31], meaning that patients with normal aortic diameter at screening at age 65 still are at risk of developing AAA [32]. Despite the relatively short time of follow-up in the present study, interestingly, two patients who underwent screening still suffered from aneurysm rupture. As rAAA is a condition associated with a high mortality and as AAA is generally asymptomatic before rupture, the disease seems well suited for screening. Though, with the increasing use of radiologic imaging in medical practice, incidental AAA findings are common [33] and might impact the cost-effectiveness of screening in an era of decreasing AAA prevalence.

In the present study, we found a high rAAA mortality rate during 2010–2014. This might be explained by the fact that over 50 % of rAAA patients were aged 80 years or older, with age-related comorbidities. We also found an increased ratio of elective AAA treatment and a lower age of elective AAA treatment in males following the start of AAA screening in males. Hence, 65-year old males with AAA were likely identified by screening and electively treated, whereas in the non-screened population above 80 years aneurysms might remain unidentified until rupture. As these findings were not seen in women, they might be a consequence of screening, even if no significant reduction in incidence of rAAA in men could be demonstrated. The total incidence of elective AAA repair has decreased significantly during the past 14 years, explained by the falling AAA prevalence during the last decade [8, 9, 26].

The study limitations includes the potential risk for type two-error in not detecting a significant reduction of rAAA, due to the relatively short time of follow-up after start of AAA screening and the low incidence of rAAA. Furthermore, data from previous study periods should be interpreted with caution, and the considerable reduction in autopsy rate especially among the elderly [34] might have biased the assessment of rAAA incidence towards an underestimation.

## Conclusions

This study confirms the reduction in the incidence of rAAA in men since the beginning of the 21st century [2, 8, 20, 22]. This might be explained by an improved control of cardiovascular risk factors and in particular decreasing smoking rates. We were not able to demonstrate a reduction in rAAA incidence 4 years after start of screening, however, and it is important to continue assessing the effects of screening in the future.
